# A Microfluidic-Based Cell-Stretching Culture Device That Allows for Easy Preparation of Slides for Observation with High-Magnification Objective Lenses

**DOI:** 10.3390/mi16010093

**Published:** 2025-01-15

**Authors:** Momoko Kato, Kae Sato

**Affiliations:** Department of Chemical and Biological Sciences, Faculty of Science, Japan Women’s University, 2-8-1 Mejirodai, Bunkyo, Tokyo 112-8681, Japan

**Keywords:** microfluidic cell-stretching device, high-magnification objective lens, YAP1

## Abstract

Microfluidic-based cell-stretching devices are vital for studying the molecular pathways involved in cellular responses to mechanobiological processes. Accurate evaluation of these responses requires detailed observation of cells cultured in this cell-stretching device. This study aimed to develop a method for preparing microscope slides to enable high-magnification imaging of cells in these devices. The key innovation is creating a peelable bond between the cell culture membrane and the upper channel, allowing for easy removal of the upper layer and precise cutting of the membrane for high-magnification microscopy. Using the fabricated device, OP9 cells (15,000 cells/channel) were stretched, and the effects of focal adhesion proteins and the intracellular distribution of YAP1 were examined under a fluorescence microscope with 100× and 60× objectives. Stretch stimulation increased integrinβ1 expression and promoted integrin–vinculin complex formation by approximately 1.4-fold in OP9 cells. Furthermore, YAP1 nuclear localization was significantly enhanced (approximately 1.3-fold) during stretching. This method offers a valuable tool for researchers using microfluidic-based cell-stretching devices. The advancement of imaging techniques in microdevice research is expected to further drive progress in mechanobiology research.

## 1. Introduction

Cellular mechanotransduction is an important biological process in living organisms, as cells in the cardiovascular, pulmonary, intestinal, and skin systems are naturally exposed to stretch stimuli. These stimuli are converted into chemical signals within the cell that help maintain tissue function by influencing cell shape, proliferation, and differentiation [1,2,3]. Therefore, assays using these cells require a system capable of applying stretch stimuli.

Polydimethylsiloxane (PDMS)-based thin membranes are used as substrates for culturing cells under stretch conditions [4]. A common device for this is the uniaxial cell stretcher, where cells seeded on the flexible PDMS thin membrane are stretched using a stepper motor connected to the side of the chamber. However, its open chamber design is not suitable for small cell samples, as closed channels are more effective in preventing splashing and drying of the culture medium.

To address the limitations of open cell-stretching culture chambers, microfluidic devices have been developed over the past decade for use in mechanobiology research [5,6,7,8,9,10,11,12]. These devices employ PDMS thin membranes as cell culture substrates, which are stretched using either motors [6,13] or air pressure [14]. Microfluidic devices that use air pressure to stretch thin membranes are smaller and more cost-effective than motorized systems.

The lung chip, the most well-known device that uses air pressure to stretch a PDMS-based porous membrane [14], has since been applied to study the complex function and pathophysiology of several human organs, including the intestine [15,16,17], kidneys [18], and liver [19]. These devices [14,15,16,17,18,19] stretch the membrane by pulling it into control chambers on either side of the cell culture channel. Furthermore, devices have been developed to stretch PDMS-based non-porous membranes by pulling them into control chambers below the cell culture channel [7,9]. These devices have been used to culture vascular smooth muscle cells and support research on progeria [7] and pulmonary hypertension [9].

The use of organs-on-chips has enabled cellular-level analysis. However, many cell-stretching microdevices have significant limitations. One drawback is that the cell culture surface is far from an objective lens, often requiring observation with a 20× objective lens rather than a high-magnification lens [20]. While high-magnification imaging, confocal [21], and SEM [20] images have been reported, such as by microtome sectioning, this approach is inconvenient. While common cell-stretching microdevices are three-layered with culture chambers above and below the PDMS-based thin membrane [22], two-layered devices without a lower chamber for close proximity between the objective lens and cell culture substrate have been reported [23]. While these devices allow for real-time high-magnification imaging, they are challenging to manufacture. Advanced manufacturing techniques are required because the joint width between the PDMS membrane and the chamber is only 100 µm. Hence, developing a more convenient method for observing cells on the membrane with high magnification is necessary.

**Figure 1:**
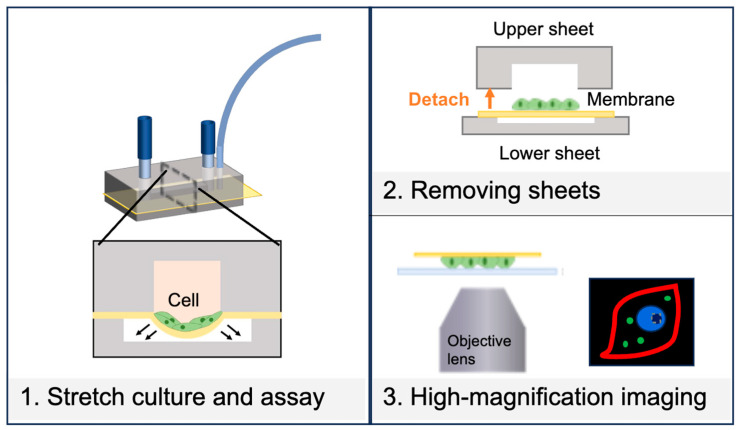
Scheme of the new cell-stretching device in this study.

This study aimed to fabricate a three-layer cell-stretching device with channels above and below the PDMS thin membrane, featuring a peelable bond between the upper channel and the membrane. Unlike previous cell-stretching devices where the PDMS thin membrane and channels were permanently bonded using plasma treatment [7,9], the newly developed device allows the upper channel to be peeled off before observation (Figure 1). This enables the direct placement of a coverslip on the cell surface for close, high-magnification imaging. With the upper channel removed, it is easier to cut out only the membrane with the cells attached. OP9 cells, which are stromal cells derived from mouse bone marrow, were cultured, fixed, and immunostained in the fabricated device. The cell culture surface was then cut out, and the cell proteins were observed under a fluorescence microscope using 100× and 60× objective lenses. The fluorescence images obtained were analyzed to determine changes in protein expression and distribution during stretch stimuli. The procedure for preparing microscope slides, including disassembling the cell-stretching device, has not been previously reported in detail, making this simple method for cell observation highly valuable for researchers. In addition, OP9 cells are used as feeder cells to induce hematopoietic differentiation [24]. Since hematopoiesis is thought to be related to blood vessel stretching [25], it is of interest whether OP9 cells respond to mechanical stimuli.

## 2. Materials and Methods

### 2.1. Fabrication of a Cell-Stretching Device

Microchannels were fabricated by molding as previously described [9,26]. A degassed PDMS mixture (SILPOT 184; Dow Corning, Midland, MI, USA) was poured to a thickness of 4 mm (upper sheet) or 1 mm (lower sheet), with upper (1 × 1 × 10 mm) or lower (the structure consists of 3 × 0.5 × 3 mm squares lined up at one end of a 2 × 0.5 × 12 mm rectangular base) channel structures, respectively, and baked at 65 °C for 1 h. The PDMS replica was adhered to a glass slide (26 × 76 mm) and baked at 100 °C for 1 h. Through-holes were created at both ends of the upper microchannel using a 2.0 mm biopsy punch (BP-20F, KAI Industries, Tokyo, Japan).

**Figure 2:**
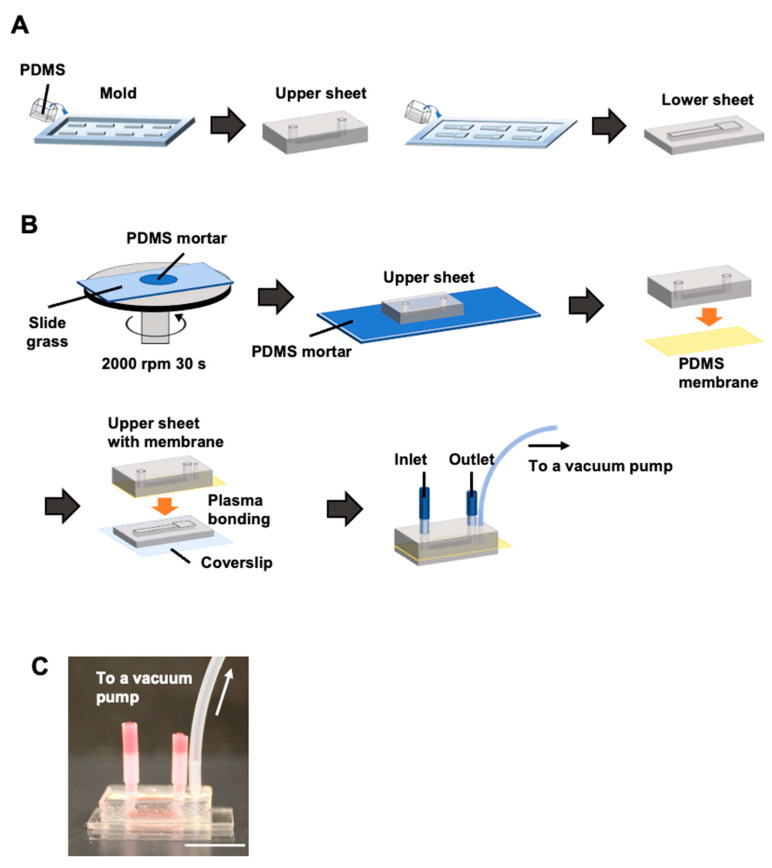
Device fabrication steps. (**A**) The upper and lower sheets were created from PDMS. (**B**) Device assembly and tubing process. The upper sheet and PDMS thin membrane were bonded using PDMS mortar, and the lower sheet and PDMS thin membrane were permanently bonded using plasma. (**C**) Photograph of the completed device. Scale bar = 10 mm.

Figure 2 shows the assembly of the cell-stretching device. The PDMS thin membrane (50 µm thickness, CF-050-5A, Mitsubishi Chemical, Tokyo, Japan), which serves as a substrate for cell attachment, was bonded to the PDMS upper sheet using PDMS mortar. Specifically, 600 µL of PDMS mortar, which is degassed PDMS mixed with hexane (Special Grade, 18041-00, Kanto Chemical, Tokyo, Japan) at a mass ratio of 1:3 to reduce the viscosity of PDMS [27], was placed at the center of a 76 mm × 52 mm glass slide and spin-coated at 2000 rpm for 30 s. After standing for 10 min, the bonding surface of the upper sheet was placed on top of the mortar and left for 5 min. The upper sheet was joined to the thin membrane of the PDMS, with one support side removed, and left to stand for 30 min. After baking at 100 °C for 1 h, the thin membrane support was removed, and a 1 mm diameter hole was punched through one side of the channel (BP-10F, KAI Industries) from the thin membrane surface to create a degassing hole.

Plasma treatment was applied at 100 W for 35 s to the bonded surfaces of the PDMS lower sheet and the thin membrane side of the upper PDMS sheet. The plasma-irradiated surfaces were then bonded, aligning the upper and lower channels with the degassing holes and square areas of the lower channel. A 1 kg weight was then placed on approximately six devices and baked at 100 °C for 1 h.

Each hole in the upper channel was connected to a polytetrafluoroethylene (PTFE) tube (1.0 mm id × 2.0 mm od × 10 mm long; Nichias, Tokyo, Japan). The PTFE tube was glued to the upper sheet with the uncured PDMS and baked at 100 °C for 1 h. The other end of the PTFE tube was connected to another PTFE tube (1.93 mm id × 2.53 mm od × 11 mm for inlet or 7 mm for outlet; AWG13, Chukoh Chemical, Tokyo, Japan).

A PTFE tube (0.5 mm id, 1.0 mm od, 10 mm long; TUF-100, Chukoh Chemical) was inserted into the degassing hole in the upper sheet without reaching the bottom of the lower channel. A silicone tube (1.0 mm id × 2.0 mm od × 100 mm long) was inserted to completely cover the PTFE tube, and the tip of the silicone tube was in contact with the surface of the upper sheet. Uncured PDMS was applied to the root of the silicone tube and baked at 100 °C for 1 h to bond the tube to the upper sheet.

### 2.2. Cell Stretch System

**Figure 3:**
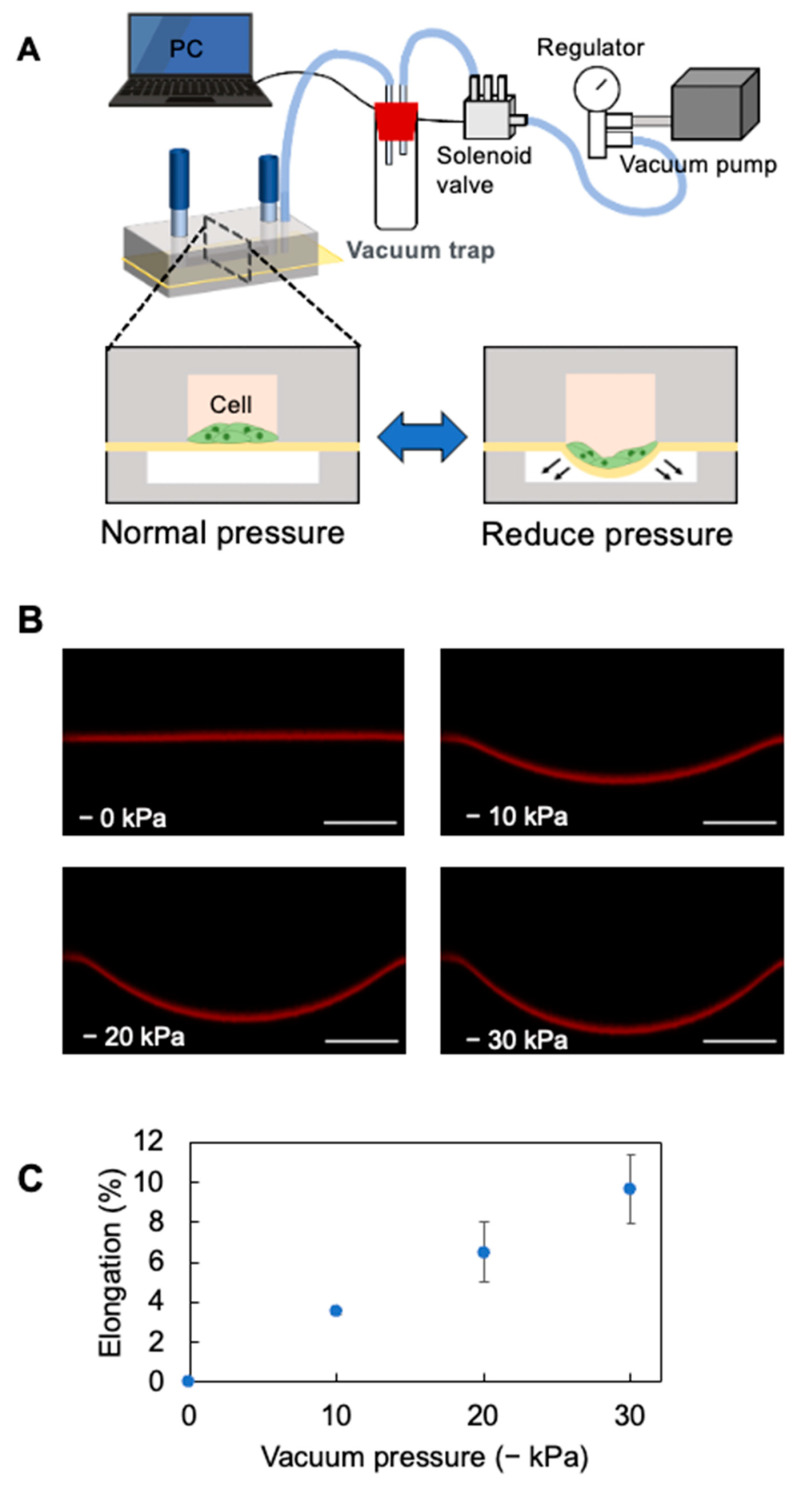
Stretched PDMS thin membrane in the device. (**A**) Setup of the stretching apparatus and device. As indicated by the arrows, a PDMS membrane was drawn in the lower channel. (**B**) Cross-section of the device reconstructed from confocal microscope images. Scale bar = 300 µm. (**C**) Graph of PDMS thin membrane elongation at different vacuum pressures. Mean ± SD, *n* = 3.

Figure 3A illustrates the microfluidic cell stretch system. The cell stretch system consists of five main parts: a device, solenoid valve, PC controlling the vacuum and normal pressure in the lower channel, vacuum pump, and pressure regulator. The silicon tube (1.0 mm id × 2.0 mm od × 100 mm long) at the lower channel was connected to the inlet of a centrifugal tube via another silicon tube (2.0 mm id × 4.0 mm od). The silicone tube (2.0 mm id × 4.0 mm od) at the outlet of the centrifuge tube was connected via a silicone tube (4 mm id × 7 mm od) to polyurethane tubing (2.5 mm id × 4 mm od; UBT0425-20-B, PISCO, Nagano, Japan) that connected to the inlet of a solenoid valve (V114-SMOU, SMC, Tokyo, Japan). A 2.5 mm id × 4 mm od polyurethane tube at the outlet of a solenoid valve was connected to a 4 mm id × 7 mm od silicone tube. The silicone tubing was connected to a regulator (RVV8UG, PISCO) via a tubing connector and polyurethane tubing (5 mm id × 8 mm od; UB850-5-C, PISCO).

The vacuum pressure was set to −30 kPa using the regulator. The solenoid valve was controlled with a NI9477 digital switch and LabView software (National Instruments, Austin, TX, USA), with each valve opening or closing every 1 s (0.5 Hz).

### 2.3. Analysis of the Stretching Properties of the PDMS Membrane

Fluorescence microscopy was used to measure the stretch ratio of the PDMS thin membranes. Rhodamine B (10 µM, 50 µL; 183-00122, FUJIFILM Wako Pure Chemical, Osaka, Japan) was introduced into the upper channel. Fluorescence images of Rhodamine B adsorbed on the membrane were then acquired using a confocal microscope, FV-1200 (Evident, Tokyo, Japan), with a 473 nm laser and a 10× objective lens (UPLSAPO 10x, Evident). The images were captured before and after vacuuming the lower channel, and the total length of the membrane was measured from the acquired z-axis stacked images using Image J (National Institutes of Health, Bethesda, MD, USA). The ratio of the length after the vacuum to that before the vacuum was calculated as a PDMS membrane stretch ratio.

### 2.4. Cell Culture in Cell-Stretching Devices

OP9 cells, which are stromal cells derived from mouse bone marrow, were used for culturing. The medium was an α-MEM solution prepared from an Alpha Medium (12000-022, Thermo Fisher Scientific, Waltham, MA, USA), with 20% fetal bovine serum (SH30910.03, HyClone, Logan, UT, USA), 1% penicillin–streptomycin (P4333, Sigma-Aldrich, St. Louis, MO, USA), and 1% L-glutamine (25030-081, Thermo Fisher Scientific). The channels were coated with fibronectin by introducing 40 µL of 50 µg/mL fibronectin solution (C-43050, Promo Cell, Heidelberg, Germany) into the device channels and incubated at 37 °C for at least 2 h. The fibronectin solution in the channel was removed, 100 µL of the medium was introduced and incubated (37 °C) for 30 min, and 50 µL of OP9 cell suspension (3.0 × 10^5^ cells/mL, 15,000 cells/channel) was slowly introduced into the channel. After 72 h of static culture, cells were incubated under cyclic stretch (0.5 Hz) or static conditions for 24 h to observe integrinβ1 and vinculin subcellular localization and for 4 h to observe YAP1 subcellular localization.

### 2.5. Cell Staining

The cells were washed three times with 100 µL of phosphate-buffered saline (PBS [+]) for 1 min each, then fixed with 100 µL of 4% paraformaldehyde for 15 min at 23 °C. Cells cultured under the cyclic stretch condition were fixed with the PDMS thin membrane extended. After washing the cells three times with 100 µL of PBS(+), the tube connected to the device was removed. Cells were permeabilized with Triton x-100 (40 µL, 0.1% *v*/*v*) for 15 min at 23 °C, followed by 40 µL of 0.5% bovine serum albumin (BSA) and blocking for 1 h at 23 °C.

For integrinβ1, vinculin, f-actin, and nuclear staining, 0.5% BSA was added with anti-integrinβ1 antibody (0.4 µg/mL; SC-19656, Santa Cruz Biotechnology, Dallas, TX, USA), anti-vinculin antibody (4 µg/mL; 26520-1-AP, Proteintech, Rosemont, IL, USA), and rhodamine phalloidin (1.65 µM; R4159, Thermo Fisher Scientific) and incubated overnight at 4 °C. The next day, cells were washed three times with PBS(+) and incubated with a mixture of secondary antibodies—Alexa Fluor 488-conjugated goat anti-Armenian hamster IgG (2 µg/mL; ab173003, Abcam, Cambridge, UK) for anti-integrinβ1 and Alexa Fluor 647-conjugated goat anti-rabbit IgG (2 µg/mL; (H + L), A21245, Thermo Fisher Scientific) for anti-vinculin in 40 µL—and stained for 1 h at 23 °C.

For YAP1 staining, 40 µL of anti-YAP1 antibody (2 µg/mL; SC-101199, Santa Cruz Biotechnology) in 0.5% BSA was added. The next day, 40 µL of secondary antibody (4 µg/mL; goat anti-Mouse IgG (H + L) Alexa Fluor Plus 647, A32728, Thermo Fisher Scientific) in 0.5% BSA was introduced for staining.

Cells were then washed three times with 40 µL of PBS(+), fixed with 40 µL of 4% paraformaldehyde at 23 °C for 5 min, and washed three times with 40 µL of PBS(+). Finally, 40 µL of Hoechst33342 (10 µg/mL; H3570, Thermo Fisher Scientific) was allowed to stand at 23 °C for 10 min, followed by three washes with 40 µL of PBS(+).

### 2.6. Microscopic Observation

**Figure 4:**
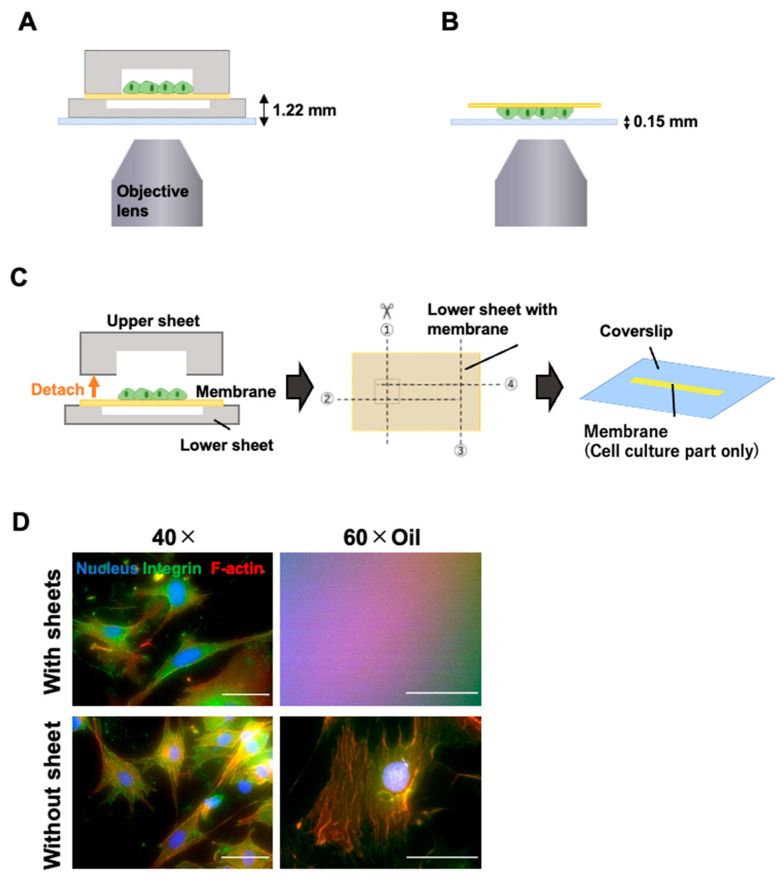
Advantages of cell-stretching culture device with a detachable channel. (**A**) Schematic of microscopic observation with the upper sheet attached to the thin membrane. (**B**) Schematic of microscopic observation after removing both sheets from the thin membrane. (**C**) Preparation for microscopic observation. The upper sheet was removed from the PDMS thin membrane, and only the cell culture area of the thin membrane was cut out and placed on the coverslip. The membrane was cut in the number order shown in the figure. (**D**) Fluorescence microscopy image of a cell observed with 40× and 60× oil immersion objective lenses. Scale bar = 50 µm.

After staining the cells, a specimen was prepared for microscopy (Figure 4). The top sheet was peeled off the PDMS thin membrane using tweezers, and only the cell culture portion of the thin membrane attached to the bottom sheet was cut out with scissors and placed, cell-culture-side down, on a 24 mm × 24 mm coverslip with a 0.6 µL drop of Slowfade (S36967, Thermo Fisher Scientific).

Fluorescence images were acquired using a microscope (IX83, Evident) equipped with a 100 W high-pressure mercury lamp and a cooled CCD camera ORCA-R2 (Hamamatsu Photonics, Shizuoka, Japan). A dichroic mirror block U-FBNA (excitation 470–495 nm, absorption 510–550 nm) was used to observe integrinβ1, U-FGW (excitation 530–550 nm, absorption > 575 nm) to observe f-actin, and U-FUW (excitation 340–390 nm, absorption > 420 nm) to observe nuclei. A 40× objective (LCPLFL 40×, Evident) and a 60× oil immersion lens (UPLSAPO 60× Oil, Evident) were used.

A confocal microscope, FV1200 (Evident), was also used to examine the intracellular distribution of integrinβ1, vinculin, f-actin, and YAP1. Lasers with 473 nm, 559 nm, 473 nm, and 405 nm were used for integrinβ1, vinculin, f-actin, and nuclei, respectively. The objectives were 60× and 100× oil immersion lenses (UPLSAPO 100× Oil, Evident). For YAP1, the 559 nm and 405 nm lasers for the nuclei, a 20× objective lens (UPLSAPO 20×, Evident), and a 60× oil immersion lens were used. The distribution of integrinβ1, vinculin, and YAP1 was analyzed using maximum-intensity projection images.

## 3. Results and Discussion

### 3.1. Elongation Rate of the PDMS Thin Membrane in the Cell-Stretching Device 

Figure 2C and Figure 3A show the completed device and stretcher setup. The thin membrane of the PDMS was stretched by pulling it into the chamber while the lower chamber was vacuumed. Rhodamine adsorbed on the PDMS thin membrane was observed using confocal microscopy to calculate its elongation rate. In the acquired z-axis stacked image (Figure 3B), the adsorbed rhodamine on the PDMS thin membrane formed an arc as the lower channel was depressurized. The PDMS mortal joint did not delaminate during the stretching process. The total cross-sectional length of the PDMS thin membrane in the uniaxial direction of the channel was then measured using this image (Figure 3B). The total length was 1.265 ± 0.001 mm at normal pressure and 1.310 ± 0.002 mm, 1.348 ± 0.019 mm, and 1.388 ± 0.022 mm at reduced pressures of −10 kPa, −20 kPa, and −30 kPa, respectively. The elongation rate of the PDMS membrane (Figure 3C) showed a 10% increase at reduced pressure (−30 kPa) compared to normal pressure. 

### 3.2. Microscopic Observation with High-Magnification Objective Lens

The cells cultured in the cell-stretching device were stained with fluorescence antibodies and then observed under a microscope. The distance between the device bottom and the cell culture surface was more than 1.22 mm (Figure 4A). Therefore, the upper and lower PDMS sheets were detached from the thin membrane to enable microscopic observation using a high-magnification objective lens (Figure 4B). The PDMS mortar bond between the upper sheet and thin membrane was easily removed, exposing the thin membrane surface with attached cells (Video S1). When only one sheet was removed, and the thin membrane was placed on the coverslip with the other sheet, the membrane wrinkled, making it difficult to focus the microscope. Based on this result, we removed both sheets and cut out only the cell culture portion of the thin membrane (Figure 4C). The upper sheet was removed, and the cell culture surface of the thin membrane attached to the lower sheet, which had a wider channel, was cut off and placed on a coverslip. Observation was possible with a 40× objective lens without removing the upper and lower sheets from the thin membrane with a long working distance (Evident, LCPLFL 40×, W.D.: 2.15–2.89 mm) (Figure 4A), but not with a 60× oil immersion objective lens with a short working distance (Evident, UPLSAPO 60× Oil, W.D.: 0.15 mm) (Figure 4D, with sheet). However, when only the thin membrane containing cultured cells was placed on the coverslip (Figure 4B), observation was possible with both lenses (Figure 4D, without sheet).

### 3.3. Effects of Cyclic Stretching on Stress Fibers and Focal Adhesion

After developing a method for preparing a microscope slide for high-magnification observation, we proceeded to use a confocal microscope. OP9 cells were periodically stimulated with the device to observe the subcellular localization of integrinβ1 [28], vinculin [29] (which are mechanosensory proteins and members of the focal adhesion protein), and f-actin (a cytoskeletal protein) [30].

**Figure 5:**
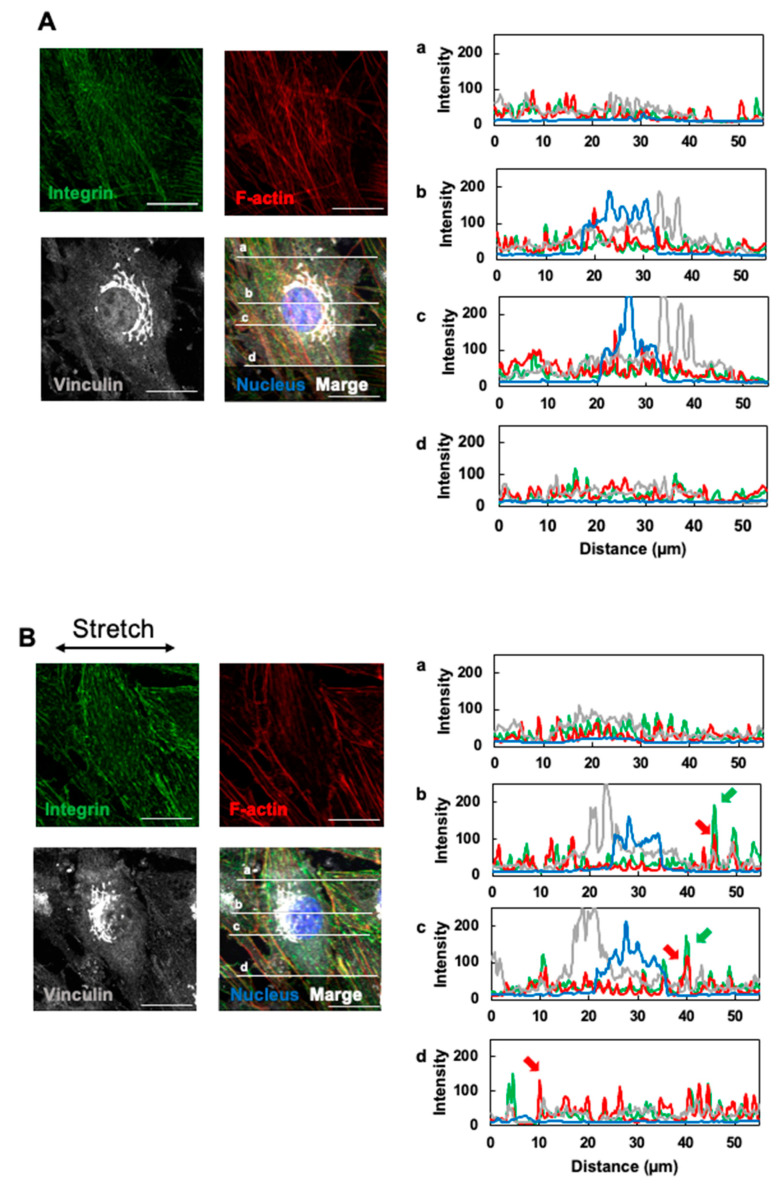
Effects of cyclic stretching on integrinβ1 and vinculin localization in OP9 cells. Fluorescence images of integrinβ1 (green), f-actin (red), and vinculin (gray) in OP9 cells cultured on the device after 72 h under static conditions followed by 24 h under (**A**) static or (**B**) cyclic stretching conditions (0.5 Hz) photographed with a 100× oil immersion objective lens. Confocal images were captured every 0.48 µm along the z-axis to create z-stacks (14 slices) for maximum-intensity projection (scanning zoom 2×). The right figure shows the fluorescence intensity–distance profiles of integrinβ1 (green), f-actin (red), vinculin (gray), and nuclei (blue) on lines (**a**–**d**) drawn on the microscope image (MIP).

Figure 5 shows fluorescence images captured using a 100× oil immersion objective lens (Evident, UPLSAPO 100× oil). In both static (Figure 5A) and stretched (Figure 5B) conditions, integrinβ1 was distributed in a striated pattern, and a portion of vinculin accumulated near the nucleus. Figure 5(Aa–Ad),(Ba–Bd) show the fluorescence intensity and distance profiles of integrinβ1, vinculin, f-actin, and nuclei on any four lines drawn on this fluorescence image for the static and stretched condition, respectively. In stretch-stimulated cells, integrinβ1 exhibited areas of high fluorescence intensity (Figure 5(Bb,Bc) green arrows), especially at the cell periphery. This suggests increased expression and accumulation, consistent with prior reports on mechanical stretch stimulation increasing integrinβ1 expression [31,32]. 

Stretch stimuli are known to increase vinculin binding to integrin-based focal adhesions [29,33]. Therefore, using the fluorescence intensity profiles in Figure 5(Aa–Ad),(Ba–Bd), we counted the fluorescence intensity peaks of vinculin at the same coordinates as the integrinβ1 fluorescence intensity peaks and compared the percentages between static and stretch conditions. The percentage of integrins co-localized with vinculin relative to these total integrinβ1 peaks was 49.5 ± 6.7% for the static condition and 67.5 ± 10.2% for the stretch condition (*p* = 0.043, *t*-test). The percentage of vinculin peaks overlapping with integrinβ1 peaks was significantly higher in the stretch condition. Stretch stimulation promoted the formation of integrinβ1 and vinculin complexes in OP9 cells. 

Regarding stress fiber formation by f-actin polymerization, which is generally enhanced by stretch stimuli [34], the fluorescence intensity increased only at the edges of stretch-stimulated OP9 cells (Figure 5(Bb–Bd), red arrows). No significant difference was observed in the rest of the cytoplasm compared to the static condition.

### 3.4. Effects of Cyclic Stretching on YAP1 Intracellular Localization

**Figure 6:**
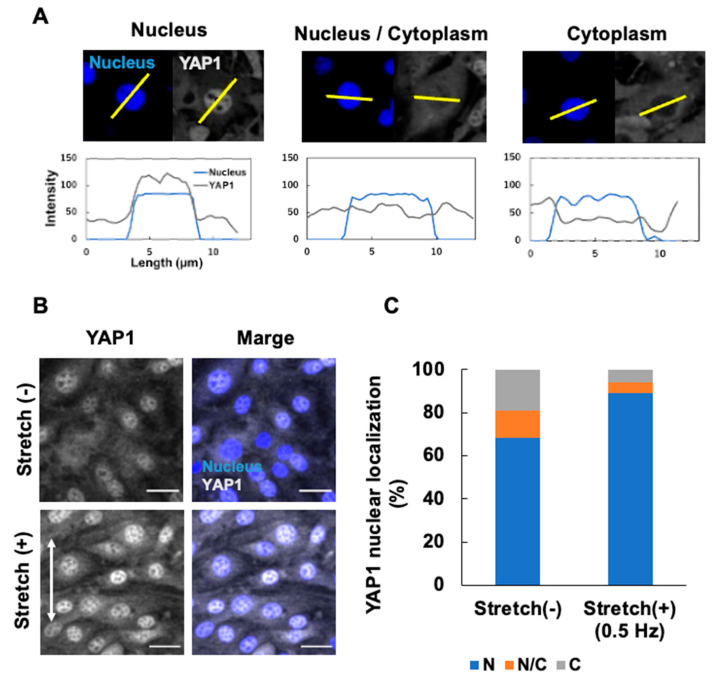
Effects of cyclic stretching on YAP1 localization in OP9 cells. (**A**) Criteria for evaluating the subcellular localization of YAP1. Cross-sections through the center of the cell nucleus were selected, and profiles of nuclear and YAP1-derived fluorescence signal intensities at the cross-sections were obtained. Three patterns were observed: cells with YAP1 localized in the nucleus, cells with YAP1 localized in both the nucleus and cytoplasm, and cells with YAP1 localized in the cytoplasm. (**B**) Fluorescence images of OP9 cells in the static condition or in response to cyclic stretching (0.5 Hz) for 4 h after 72 h of static culture. Confocal images were acquired every 0.51 µm along the z-axis to create z-stacks (35 slices) for maximum-intensity projection. Scale bar = 30 µm. (**C**) The percentage of cells with YAP localization in the nucleus under the culture conditions is shown in (**B**), with an average of four microscopy images taken from four different devices. N = nucleus. C = cytoplasm. *p* = 0.013, * *p* > 0.05.

After several hours of stretch stimulation, YAP1 translocated to the nucleus, playing a role in regulating cell proliferation and differentiation by activating transcription [35,36,37]. The localization of YAP1 and nuclei in OP9 cells stretched for 4 h was observed. Based on the fluorescence intensity graph generated by FLUOVIEW ver 4.2 Viewer (Evident), the subcellular distribution of YAP1 was classified into three patterns: nuclear, nuclear and cytoplasmic, and cytoplasmic (Figure 6A). OP9 cells cultured under static or stretched conditions (0.5 Hz) were classified into these three patterns, and the percentage of each pattern among all classified cells was calculated (Figure 6B). For each static and stretched condition, four fluorescence images with approximately 65 cells (cell density: 590–690 cells/mm^2^) were analyzed to calculate the average number of cells. The number of cells localized in the nucleus was 45.5 ± 5.1 and 58.0 ± 2.1; in the nucleus and cytoplasm, it was 8.5 ± 3.6 and 3.5 ± 1.8; and in the cytoplasm, it was 12.8 ± 2.2 and 3.8 ± 0.8 for the static and stretched conditions, respectively (Figure 6B). The percentage of OP9 cells localized to the nucleus was 89% in the stretched condition and 68% in the static condition (*p* = 0.00442 < 0.005, *t*-test), consistent with previous reports [35,36,37] showing increased nuclear localization with stretching. 

## 4. Conclusions

In this study, we developed a microfluidic cell-stretching device with an easily detachable upper microchannel. Table 1 shows the comparison between our device and other technologies. By removing the upper sheet and isolating the membrane with the attached cells, we created a slide suitable for observation under a microscope with a high-magnification lens. OP9 cells were stretched using the device, and the effects on focal adhesion proteins and the intracellular distribution of YAP1 were examined using a fluorescence microscope with 100× and 60× objectives.

This method is useful for users of microfluidic-based cell-stretching devices. By cutting out the membrane, it can be used for both fluorescence and electron microscopy without the use of a microtome. Similar to previous devices, our device can also simultaneously perform flow culture and stretch culture. As observation methods advance, studies using microfluidic-based cell-stretching devices will be warranted in the future.

## Figures and Tables

**Table 1:** Comparison of device performance.

	Use of High-Magnification Lens	Ease of Device Fabrication	Combination with Flow Culture
This work	✓	✓	✓
Mao, 2021 [22]	✓	−	✓
Grassart, 2019 [20]	Possible with microtome.	−	✓

## Data Availability

The original contributions presented in the study are included in the article. Further inquiries can be directed to the corresponding author.

## Supplementary Materials

The following supporting information can be downloaded at https://www.mdpi.com/article/10.3390/mi16010093/s1. Video S1: Upper channel peeling process.
